# A rare case of sinonasal solitary fibrous tumour in a patient with concurrent papillary thyroid carcinoma: A case report

**DOI:** 10.1016/j.amsu.2021.103032

**Published:** 2021-11-10

**Authors:** Nurul Anis Mohd Fauzi, Noor Idayu Ibrahim, Wan Faiziah Wan Abdul Rahman, Sharifah Emilia Tuan Sharif, Muhammad Nasri Abu Bakar, Ramiza Ramza Ramli

**Affiliations:** a Department of Otorhinolaryngology-Head & Neck Surgery, School of Medical Sciences, Universiti Sains Malaysia, Health Campus, 16150, Kubang Kerian, Kelantan, Malaysia; b Hospital Universiti Sains Malaysia, Health Campus, 16150, Kubang Kerian, Kelantan, Malaysia; c Department of Pathology, School of Medical Sciences, Universiti Sains Malaysia, Health Campus, 16150, Kubang Kerian, Kelantan, Malaysia; d Department of Otorhinolaryngology-Head & Neck Surgery, Hospital Sultan Ismail Petra, KM 6, Jalan Kuala Krai – Gua Musang, 18000, Kubang Kerian, Kelantan, Malaysia

**Keywords:** Case report, Solitary fibrous tumour, Paranasal sinus neoplasm, Immunohistochemistry

## Abstract

**Introduction and importance:**

A solitary fibrous tumour (SFT) is a rare neoplasm that commonly arises in the pleura and can occur in other extrathoracic sites. Extrapleural SFT, particularly in the sinonasal cavity, is extremely rare. There are no definite diagnostic criteria for sinonasal SFT as it is rare. Histologic analysis with immunohistochemistry plays an important role in diagnosing SFT.

**Case presentation:**

We report herein a case of SFT of the sinonasal cavity, which later spread to the oral cavity in a 67-year-old male with underlying papillary thyroid carcinoma (PTC) stage IV. He complained of recurrent epistaxis from a mass in his left nasal cavity for two weeks. The mass grew bigger, and spread to the oral cavity, causing dysphagia and upper airway obstruction. Tracheostomy was done under local anaesthesia and a biopsy of the mass was taken to rule out metastasis from the PTC. However, histopathological examination revealed a mesenchymal tumour of fibroblastic type, consistent with an SFT. He was planned for surgical resection of the tumour. However, he refused the operation and was lost to follow-up.

**Clinical discussion:**

We describe the clinical presentation of this rare tumour of the sinonasal and oral cavity, including upper airway obstruction, and the importance of immunohistochemical markers such as CD34 and BCL-2 in diagnosing SFT. Complete resection of the tumour is the definitive treatment for SFT.

**Conclusion:**

SFT of the sinonasal and oral cavity is extremely rare. Upper airway obstruction may occur due to the location of the tumour in the airway region. Immunohistochemistry is crucial to distinguish this tumour from other mesenchymal tumours.

## Highlights

• Solitary fibrous tumour (SFT) in the nasal cavity and paranasal sinus is extremely rare. In our case, the tumour had spread to the oral cavity, causing upper airway obstruction.
• Urgent trachestomy is needed in view of upper airway obstruction due the massive tumour in the upper airway.
• Our patient also had double pathology, which was SFT of the sinonasal cavity with underlying papillary thyroid carcinoma (PTC) stage IV. Thus, we had to rule out metastasis from PTC to the sinonasal cavity.
• Immunohistochemical studies are the most sensitive and specific method for diagnosing an SFT. Among the most sensitive first-line markers are CD34, BCL-2 and STAT6.

## 1 Introduction

A solitary fibrous tumour (SFT) is a rare mesenchymal tumour and makes up only 2% of all soft tissue tumours [1]. It usually originates from the pleura and other intrathoracic or peritoneal locations [2]. However, it may also arise from other anatomic locations. SFT is found in around 5–27% of reported cases in the head and neck region, mainly in the orbit and the oral cavity [3]. SFT of the sinonasal region is extremely rare and only accounts for <0.1% of all sinonasal neoplasms. The majority of them present as slow-growing masses with indolent behavior and can be fully cured by complete surgical excision [4]. SFT of the sinonasal region could be hard to distinguish from other mesenchymal lesions due to its relative rarity and various types of morphologic appearance. In our case, the patient had a concurrent sinonasal and oral cavity tumour with underlying papillary thyroid carcinoma (PTC). Hence it was important to rule out metastasis from PTC to the sinonasal and oral cavity. Biopsy was taken from the nasal and oral cavity mass and sent for immunohistochemistry analysis which was essential for accurate diagnosis. This case report has been reported in line with the SCARE Criteria [5].

## 2 Case presentation

A 67-year-old Malay male who worked as a rubber tapper presented with a two-week history of recurrent left epistaxis. He also complained of left-sided nasal blockage over the past 30 years, worsening over the past eight months. He started to notice a mass in his left nasal cavity eight months ago, associated with anosmia and ageusia. The nasal mass spread to the oral cavity, causing shortness of breath and dysphagia. He also had progressive left cheek fullness and left eye proptosis for four months. Nevertheless, his left eye vision was good, and there was no diplopia. He was already diagnosed with papillary thyroid carcinoma with spine metastasis the previous year, and was planned for a total thyroidectomy at that time, however, he refused surgical intervention. He did not take any drugs and did not have any allergy histories. He was an active cigarette smoker but did not take alcohol. Family history was unremarkable.

**Fig. 1:**
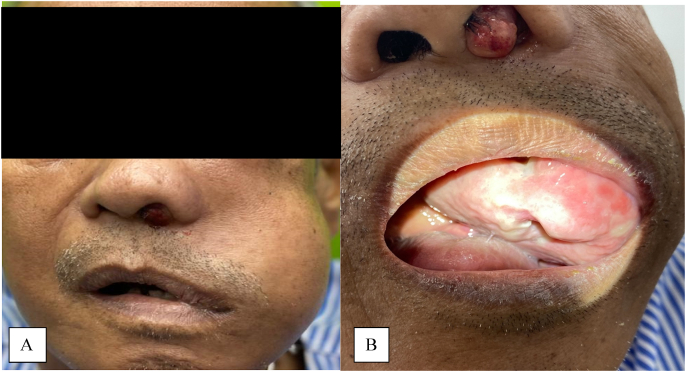
A, Fleshy mass seen protruding from the left nasal cavity, with left cheek swelling and left eye proptosis. B, Exophytic mass seen arising from the left upper gum, extending posteriorly to the soft palate.

On physical examination, a fleshy mass was seen protruding from the left nasal cavity, with left cheek swelling and left eye proptosis (Fig. 1A). Intraorally, there was an exophytic mass seen arising from the left upper gum, extending posteriorly to the soft palate (Fig. 1B). There was also a fleshy mass seen at the posterior pharyngeal wall extending from the nasopharynx. Nasoendoscopy showed fleshy mass occupying both nasal cavities. In addition, multiple matted neck nodes were found on the right side of the neck at cervical level II to IV. Clinically, he was initially diagnosed as sinonasal carcinoma with upper airway obstruction, with differential diagnosis of metastasis from papillary thyroid carcinoma. Tracheostomy was immediately done by the otorhinolaryngologist under local anaesthesia to relieve upper airway obstruction.

**Fig. 2:**
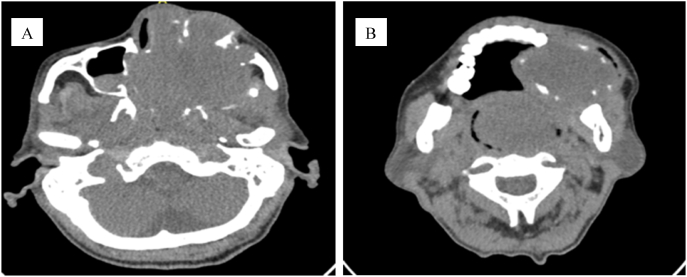
Computed tomography (CT) scan of the paranasal sinus and neck. A, Heterogenous opacification of the left maxillary sinus, bilateral nasal cavities, bilateral ethmoid, sphenoid, and frontal sinuses, extending to the nasopharynx. B, Soft tissue density extended inferiorly to the alveolar ridge of the left maxilla.

Computed tomography (CT) scan of the paranasal sinus and neck revealed heterogenous opacification of the left maxillary sinus, bilateral nasal cavities, bilateral ethmoid, sphenoid, and frontal sinuses (Fig. 2A). The soft tissue extended to the nasopharynx posteriorly and inferiorly to the alveolar ridge of the maxilla (Fig. 2B). There was an obliteration of medial extraconal space of the left orbit causing proptosis of the left eye and displacement of the left optic nerve.

**Fig. 3:**
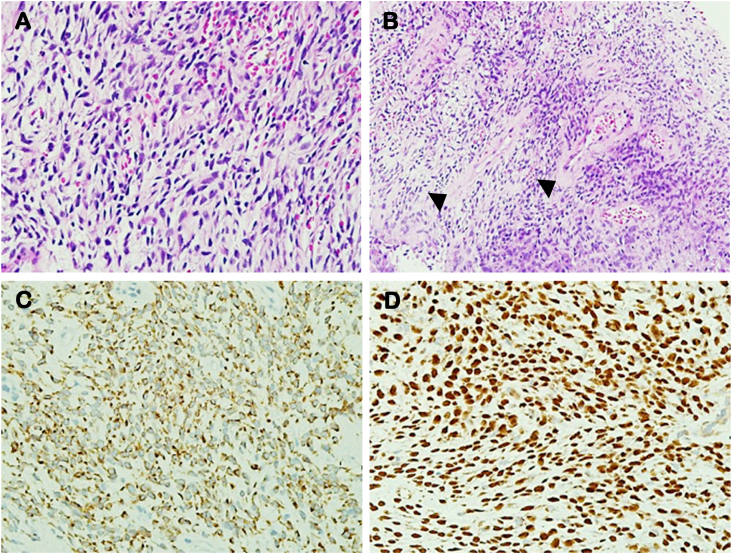
Histopathological examination findings. A, The cells exhibited spindled to ovoid nuclei with mild pleomorphism, dense chromatin pattern, eosinophilic cytoplasm and indistinct cytoplasmic borders (H&E stain, 400x). B, The stroma was loosely collagenized, with hyalinized blood vessels (arrowheads) (H&E stain, 400x). The immunohistochemistry showed diffuse, strong positive reactivity for BCL2 (C) and STAT6 (D) that support the diagnosis of solitary fibrous tumour (400x).

Histopathological examination of the left nasal and gum mass showed tumour tissue composed of haphazardly arranged spindle-shaped cells of variable cellularity and spindled to oval nuclei with mild pleomorphism (Fig. 3A). There was occasional mitotic activity detected, which was 2 mitotic figures in 10 high power fields. The tumour cells were embedded within loose collagenous stroma with hyalinized blood vessels (Fig. 3B) with some areas exhibiting stellate to staghorn blood vessels and foci of tumour necrosis. The immunohistochemical (IHC) studies showed diffuse positivity for BCL-2 (Fig. 3C), STAT6 (Fig. 3D) and focally positive for CD34. The tumour was negative for S100, cytokeratinAE1&AE3, desmin, smooth muscle actin and cyclin D1. The tissue morphology and immunohistochemistry supported a diagnosis of a solitary fibrous tumour. According to the risk stratification of Demicco system, the patient had an intermediate risk for metastasis (10% risk of metastasis at 10 years).

A multidisciplinary team discussion was held between otorhinolaryngologist, oral and maxillofacial surgeon, ophthalmologist, radiologist and pathologist, and the patient was planned for surgical resection of the tumour. He was referred to a dietician for optimization of nutrition and to an anaesthetist for pre-operative assessment. However, he sadly refused the operation and was lost to follow-up.

## 3 Discussion

SFT is a mesenchymal tumour that is mostly found in the pleura and the thoracic cavity. The extrapleural SFT rarely occurs in the head and neck area, only around 5–27%, mainly in orbit and the oral cavity [3]. SFT is extremely rare in the sinonasal region, as it only accounts for <0.1% of all sinonasal neoplasms [4]. World Health Organization (WHO) has classified SFT as a fibroblastic neoplasm with intermediate (rarely metastasizing) behavior [6]. It affects both males and females equally and generally occurs in adults in their third to the fourth decade of life, with extremes ranging from 9 to 86 years [7].

SFT is usually painless and gradually increases in size, therefore, the patient is often not aware of its presence until it has grown enough to cause symptoms and signs. Most patients with SFT of the sinonasal cavity presented with nasal blockage and other symptoms such as rhinorrhea and epistaxis [3]. Some complained of proptosis, epiphora and blurring of vision, which is due to orbital involvement of the tumour. When SFT developed in the oral cavity, the patients usually complained of dysarthria or masticatory problem [8]. Our patient also had shortness of breath due to upper airway obstruction, requiring urgent tracheostomy. To our knowledge, there were no reported cases of SFT of the sinonasal cavity that spread to the oral cavity in the past.

SFTs are macroscopically well-circumscribed and partially encapsulated with a multinodular, whitish, firm cut surface [9]. Microscopically, SFTs consist of ovoid to spindled cells with patternless growth or a storiform pattern against a collagenous background stroma with thin-walled large branching, “staghorn-shaped” blood vessels [9]. SFTs of the sinonasal region are rarely malignant. Among all the sites, the incidence rate of histologically malignant SFT is merely 10–15% [7]. Common criteria for malignant SFTs histologically are defined by the presence of nuclear atypia, necrosis, increased cellularity, ≥4 mitoses/10 HPF [7]. Demicco et al. described patient age, size of the tumour, and mitotic rate to be linked with time to metastasis and tumour-related death, and necrosis to be a predictor of metastasis [10]. In our case, the patient has intermediate risk, which is a 10% risk of metastasis at 10 years.

Immunohistochemical studies are the most sensitive and specific method for diagnosing an SFT. CD34 and BCL-2 are the most sensitive first-line markers for SFT diagnosis [2]. However, these markers have limited usefulness as they are also expressed in other neoplasms closely resembling SFT histologically [11]. CD34 expression has been shown in 81–95% of SFTs cases, but its expression is lost, especially in malignant and dedifferentiated tumours [9]. BCL-2 is more sensitive (>90% sensitivity), whereas CD99 is less sensitive (∼75%) [9]. STAT6 IHC stain has become a useful surrogate marker of NAB2-STAT6 gene fusion with remarkable sensitivity and specificity and is also expressed in malignant cases [12]. In our case, the tumour cells are positive for BCL-2, CD34 and STAT6.

SFTs have isoattenuation with the surrounding muscles and soft tissue on computed tomography (CT) imaging. The attenuation increases with intravenous contrast administration [2]. CT scan is important to assess tumour extension and bone resorption. In contrast, magnetic resonance imaging (MRI) is used to assess orbital or intracranial extension. MRI shows intermediate signal intensity on T2W1 images [13]. SFTs are highly vascular and therefore shows avid enhancement on CT and MRI images [14].

The gold standard of treatment of SFT is surgical resection with negative margins [13]. Endoscopic excision is the preferred surgical approach for sinonasal SFT, other than lateral rhinotomy, medial maxillectomy, external ethmoidectomy and transfacial endoscopic approaches which have also been described [15]. Completeness of the surgical resection determines the prognosis. Recurrence may occur in cases completely removed, probably due to difficulty achieving clear surgical margins. Tumours that cannot be eradicated or exhibit malignant histological features may respond to radiation and chemotherapy [13]. In contrast to tumours found in other sites, those in the head and neck region may impose anatomic challenges, as they have proximity to neurovascular structures and distinct anatomic compartments, such as the orbit. The prognosis of SFTs is generally good and relies on the completeness of the surgical resection.

Literature has reported synchronous occurrence of SFT with another tumour, including papillary thyroid carcinoma (PTC), pituitary macroadenoma [16], and breast carcinoma [17]. In our case, concurrent sinonasal tumour with PTC triggers the need to exclude metastasis. Therefore, immunohistochemistry analysis is crucial to making an accurate diagnosis. In this context, >90% of SFT express CD34 and STAT6, while a smaller percentage of tumours may be positive for CD99 (70%), EMA (20%), smooth muscle actin (20%) and BCL-2 (30%) [18].

In terms of prognosis, a risk stratification system for predicting metastatic risk has been recently introduced, which incorporates four variables: patient's age, numbers of mitoses, tumour size, and tumour necrosis [19]. In a validation study of the risk stratification system, the low-risk group had no metastasis, whereas the intermediate and high-risk groups conferred a metastatic risk of 7–10% in 10 years and 49–73% in 5 years, respectively [20]. In our patient, he was stratified as an intermediate-risk.

## 4 Conclusion

SFTs of the head and neck region is rare, especially in the sinonasal region. The most common presenting symptoms are nasal blockage and epistaxis. Upper airway obstruction may occur in an extensive tumour involving the sinonasal and the oral cavity, needing tracheostomy. Immunohistochemical markers are essential in diagnosing SFT, in which CD34, BCL-2 and STAT6 are the most sensitive. The definitive treatment for SFT is complete resection, and its prognosis is generally favourable and mainly depends on the completeness of tumour resection.

## Ethical approval

Not applicable.

## Sources of funding

None.

## Author contribution

Nurul Anis: data collection and design of the study, drafting the article. Noor Idayu, Muhammad Nasri: data collection. Wan Faiziah, Sharifah Emilia, Ramiza Ramza: drafting the article and revising it critically for important intellectual content, final approval of the version to be submitted.

## Informed consent

Written informed consent was obtained from the patient for publication of this case report and accompanying images. A copy of the written consent is available for review by the Editor-in-Chief of this journal on request.

## Research registration

Not applicable.

## Guarantor

Nurul Anis Mohd Fauzi.

## Provenance and peer review

Not commissioned, externally peer-reviewed.

## Declaration of competing interest

None.

## Annals of medicine and surgery

The following information is required for submission. Please note that failure to respond to these questions/statements will mean your submission will be returned. If you have nothing to declare in any of these categories then this should be stated.

### Registration of research studies

In accordance with the Declaration of Helsinki 2013, all research involving human participants has to be registered in a publicly accessible database. Please enter the name of the registry and the unique identifying number (UIN) of your study.

You can register any type of research at http://www.researchregistry.com to obtain your UIN if you have not already registered. This is mandatory for human studies only. Trials and certain observational research can also be registered elsewhere such as: ClinicalTrials.gov or ISRCTN or numerous other registries.

1. Name of the registry:
2. Unique Identifying number or registration ID:
3. Hyperlink to your specific registration (must be publicly accessible and will be checked):

### Guarantor

The Guarantor is the one or more people who accept full responsibility for the work and/or the conduct of the study, had access to the data, and controlled the decision to publish.

Nurul Anis Mohd Fauzi.
